# Knowledge, Utility, and Preferences for Beef Label Traceability Information: A Cross-Cultural Market Analysis Comparing Spain and Brazil

**DOI:** 10.3390/foods10020232

**Published:** 2021-01-23

**Authors:** Danielle Rodrigues Magalhaes, María del Mar Campo, María Teresa Maza

**Affiliations:** 1 Department Animal Husbandry and Food Science, Instituto Agroalimentario IA2, Universidad de Zaragoza-CITA, Miguel Servet 177, 50013 Zaragoza, Spain; marimar@unizar.es; 2 Department Agricultural Science and Natural Environment, Instituto Agroalimentario IA2, Universidad de Zaragoza-CITA, Miguel Servet 177, 50013 Zaragoza, Spain; mazama@unizar.es

**Keywords:** beef, traceability system, marketing, consumer, safety food, cross cultural study, questionnaire

## Abstract

The consumer environment determines consumers’ buying behavior and product preferences, and understanding these factors allows businesses in the industry to identify market demands. In view of the different contexts, Spain and Brazil, there are differences in the consumption of beef, in the production and the regulatory process concerning beef, and in particular the traceability system. The traceability system is mandatory in Spain and voluntary in Brazil. From these prerogatives, this cross-cultural study carried out through a self-administered questionnaire with 2132 Spanish and Brazilian beef buyers/consumers, aimed at comparing and understanding the familiarity with the bovine traceability system and traceability information of the label as a food security indicator. It is concluded that traceability information is well received by consumers as an attribute of credibility, and consumers are interested in ensuring that the item they buy is of known and reliable origin. But more incentives may help clarify the advantages of purchasing food with certified traceability, making it more effective for consumers to use this knowledge.

## 1. Introduction

Consumer purchasing behavior with regard to food products has been extensively studied. The context in which food products are purchased, the use of the product and the level of knowledge handled, i.e., the environment of consumption (socio-cultural, economic, technological, and political), define consumer behavior and preferences for products and processes influencing their choice at the time of purchase. It is a challenge for companies in the sector to consider all these factors, as consumer demand ultimately drives the continued investment of companies in research and innovation [1,2,3,4,5].

In view of the different contexts which, as already described, define the preference of the consumer for a particular product, the present work has been carried out in two countries, Spain and Brazil, in which there are differences in the consumption of beef, and in the production and in the regulatory framework which involves it, particularly with regard to food safety issues and with reference to traceability. The key focus of this analysis is on the fact that traceability is mandatory in Spain following European Union (EU) regulations and voluntary in Brazil [6,7,8,9,10].

The implementation of product traceability is mainly due to the food crises of the 1990s, particularly those caused by the appearance of bovine spongiform encephalopathy (BSE). Consumer trust was affected at that time, during which traceability systems proved to be inefficient, and a series of regulatory reforms were implemented, first in European countries, and later spread to other continents [11,12,13,14].

There is a great economic loss in any crisis facing the production system due to the massive loss of the product, in addition to the bad reputation suffered by the companies involved, which also creates an additional cost to regain consumer confidence in the product again [15]. Because of this set of guiding factors, traceability in this situation helps to improve the efficiency of internal management in terms of knowledge available on products and processes, which provides effective control of the food supply chain [14,16].

Traceability can be used as a method to comply with regulations and comply with food safety and quality standards, providing information on the origin, processing, and final destination of food to be related between producers and consumers [12,13]. It is also expected that if farmers and agro-food companies comply with legal traceability criteria, the role of health control would be facilitated. The more accurate the monitoring system, the faster it is possible to identify a producer and solve food safety or quality issues [17,18].

Product traceability is also important in a wider range of ideas because of the globalization of food marketing, as a result of the long distances that food travels from its origin to the consumer. Food monitoring is extremely important at this stage to discover the origin and authenticity of the food [19].

According to the activity or direction in which information is passed through the food chain, traceability can be classified into three types [20,21,22,23]:

- Internal traceability or process traceability: It is the ability to internally track the origin of products coming into the supply chain or leaving the company to control it. When using this traceability system, the main objective of industries is to enhance institutional management.
- Return traceability or supplier traceability: It is the ability of this system to find the origin and characteristics of a product based on one or more criteria at each point in the supply chain, allowing for effective product identification and management of quality and safety standards.
- Direct traceability or consumer traceability: It helps the consumer to establish trust, that is, to increase the credibility of purchased products. Foods must have a transparent tracking accompanied by information about their origin on product labels. The key theme discussed in this paper was this last example of traceability listed, which refers to traceability for consumers.

Traceability is necessary for verification of credence attributes such as origin. In general, it is considered that color, price and freshness of meat are search attributes, due to the fact that they are known before purchase, whilst taste and tenderness are experience attributes because they are only known after consumption. However, the greatest problem arises in the case of credence attributes, that is, those attributes that cannot be known even after having consumed the product or, on occasions, those with a high cost due to the adverse effects that may cause on the consumer [24]. Amongst these, animal welfare and environmentally friendly production methods, food safety, or origin can be found [25]. Grunert et al. [26] indicated the growing interest in the role of credence attributes play in consumer choice. In a recent study [27], traceability is considered a sustainability attribute like animal welfare and effect of greenhouse gas emissions.

From the literature reviewed, it is clear that traceability is highly valued by consumers for various reasons, since it is essential to know the origin of the animal and the places where the transformation processes have taken place [28]. The region of production or origin are some of the aspects most valued by consumers [29,30,31]. Many consumers show more confidence in meat produced in certain places precisely because they consider it safer. In the study of Loureiro and Umberger [28], indication of origin may only become a signal of enhanced quality if the source of origin is associated with higher food safety or quality. In this sense consumers value of country of origin depending on the number of other credence attributes included in product descriptions and the location of the consumer [32].

Therefore, traceability or certification of origin can only be used as an indication of consumer quality if it is associated to greater security. Security differs from many other attributes of quality, since it is a hard attribute to observe. A product may appear to be of high quality (i.e., color, texture, and flavor) but may not be healthy because it may be contaminated with undetected pathogens, toxic chemicals, or other health risks [13,28]. It performs in such a way that a tracked product can be considered of superior quality to a non-tracked product as a factor of competition in the industry [33].

Two lines of thinking are observed in investigations carried out on consumer confidence in traceability information: One in which it is argued that one of the ways to guarantee food safety comes from traceability, and the other in which the presence of traceability information on the labels does not translate into stronger consumer confidence.

Traceability, on the one hand, is used as a food safety method that provides product recall to identify the source of a problem [34,35]. It also acts as proof of the authenticity of the food and that the labels present in the packaging of meat products are capable of enhancing the consumer’s well-being by improving the understanding of the origin of the product, indicating that the food has been checked during the manufacturing process [28,36,37]. The certification of the product makes the consumer sure that the product is from where it actually mentions on the label [13].

On the other hand, despite the recognized need for clear information on the quality of the entire food chain, supported by modern methods of monitoring and tracking, other quality attributes may compete with the label’s traceability information. Such information, that may have little or no priority at the time of purchase, must be taken into account in any assessment of consumer preferences with regard to the certification of origin [38].

Furthermore, food quality is related to a proactive policy of creating requirements for the maintenance of a healthy food supply. However, the traceability information available on the labels does not always contribute to greater confidence, as the safety of a product is the responsibility shared by all actors in the food chain, including governments, industry and consumers, and is susceptible to failure [39].

Traceability system proponents believe that labels serve the right of the consumer to know about the origins of food products. Opponents are of the opinion that the labeling law is a protectionist measure, supported by distorted expectations of the quality of products imported. They find the labeling process expensive and complicated [40].

Amidst so many reflections on the advantages or disadvantages of traceability, do consumers know what the system of traceability is? Is this information, as it is currently shown, useful and relevant when deciding to buy beef? Do consumers trust the traceability information on the labels? Whenever necessary, do consumers know how to use this information (in a time of crisis, for example)? When a system is obligatory or voluntary, are there differences?

Communication is the crucial point of understanding between consumers and companies [38]. Once we know the types of information that different types of consumers require, we can begin to investigate whether the provision of specific information that is available through traceability will ultimately influence consumer confidence or not in making the final purchase decision [34].

Appropriate measurement methods are required to answer these questions in order to assess the value that consumers attach to information on traceability, but also to inform industry about market demand [18]. This would have significant consequences for public policy formulation [40]

The fact that the traceability system is mandatory in one country (Spain) and not in another (Brazil) could be the source of some differences in consumer perception. The purpose of this study was to understand the familiarity of Spanish and Brazilian consumers with the bovine traceability system and the label traceability information as an indicator of product safety in countries with different beef consumption, production and mainly different scale of the traceability system’s implementation.

## 2. Materials and Methods

### 2.1. Experimental Overview

In general, meat consumption differs between regions due to different dietary patterns, income levels, and product availability [41]. This statement can be extended to places like Spain and Brazil, the focus countries of this study, which show differences in terms of consumption, production, and the traceability system of beef.

With regard to beef cattle production, Spain, considered one of the largest exporters both within and outside the EU, is of great importance at the European level [6]. In Brazil, on the other hand, beef production is of global importance, with the nation ranked among the world’s largest producers and exporters of beef [8]. As far as consumption is concerned, three times more beef is consumed in Brazil than in Spain. The apparent consumption in Spain is 12.7 kg/inhabitant/year [7], while in Brazil the consumption of beef is basic and high (35.8 kg/inhabitant/year) and is the fifth largest user of this commodity worldwide [42].

The traceability of beef is another very important differential point between the two countries and the main theme of this study. In Spain, the mandatory traceability legally obligated is protected by the European framework [9]. On the opposite, in Brazil, where there is a voluntary traceability of beef [10], with the exception of the production of animals and products exported to countries requiring traceability. There is also a difference in the traceability information on the beef label that reaches the consumer in Spain and Brazil, in Spain this information is mandatory and required by law [43], while in Brazil this information is optional [10].

### 2.2. Consumers

The study was performed in 2016, with Spanish and Brazilian consumers reaching a total of 2132 regular beef purchasers. In Spain, 436 questionnaires were applied in the province of Zaragoza, and in Brazil, the questionnaires were administered in four different States—Minas Gerais (*n* = 424), Sao Paulo (*n* = 456), Parana (*n* = 406) and Santa Catarina (*n* = 410). Zaragoza was chosen because it is considered a model region in market studies in Spain due to its size and consumer behavior. In Brazil, the four regions were chosen because differ in consumption and beef production, complementing themselves to become representative of the country. The consumption of beef in the State of Minas Gerais is one of the lowest in the country, consumption in São Paulo is below the national average, in the State of Paraná is within the average and in Santa Catarina is above the average consumption. In relation to beef production, the State of Minas Gerais is the second largest producer of beef in the country, São Paulo is in ninth place, Paraná in tenth and Santa Catarina in thirteenth. The States of São Paulo and Minas Gerais are two of the main States where the largest meat processing plants in the country are gathered.

### 2.3. Online Questionnaire

The data of this study were collected through a self-administered questionnaire for dissemination in network designed with the support of the *Google.forms* software (Supplementary Materials, Web Application - Google Platform). Questionnaires were sent in each country’s native language (Spanish or Portuguese).

Two types of non-probabilistic sampling were used for data collection: Chain sampling, used to classify subjects with specific characteristics, beef consumers in our case; and conventional sampling, in which subjects are selected for their accessibility [44]. The analysis has been planned to achieve descriptive and empirical goals. Closed questions with two or more alternatives and/or scales of responses were used for this [45].

The questionnaire initially presented questions about the knowledge and correct definition of the traceability concept, the variables that make up the current traceability system, and the level of credibility and importance of the traceability system for beef.

Four additional questions/topics were then dealt with, two of which were addressed to the Spanish consumer and two to the Brazilian consumer relating to purchasing beef with traceability information or Certificate of Origin (CoO). Spanish consumers were questioned if they had taken the traceability information on meat labels into account at the time of purchase. Brazilian consumers were asked if they had already purchased beef with traceability information on the label, since traceability information is not present in all products. As the response, positive or negative, was asked to justify the answer.

The other two questions were related to the importance of traceability or CoO information on labels. Six label models (Appendix A) with a different layout and traceability information set have been suggested for Spanish consumers. Participants were asked to order the labels from the most preferred to the least preferred from the layout and set of traceability information on each of the labels.

Each label contained the same “basic” product information such as the name of the product, the category, the recommendations for cooking, the expiration date, the price, etc. But with regards to the traceability information itself, three types of data were combined between the tags:

- “Traceability” includes the mandatory information provided by law: Traceability number of the animal, animal birthplace, animal fattening location, slaughterhouse, and cutting and boning room.
- Extra information, also referred to as “plus” in this study, was added to the label with mandatory traceability information. The breed and sex of the animal and the date of birth were included
- A “QR Code” (Quick Response Code) used on the labels, in this case, for the electronic reading of the traceability information of the product for sale.

Two types of labels (Appendix B) were sent to Brazilian consumers, one of which provided traceability information commonly found on beef labels sold in Brazil, i.e., an animal traceability number and a QR code for online access to animal origin information—CoO. And another label included traceability information on the label, just like the one supplied on the labels of beef sold in Spain, i.e., animal traceability number, animal birthplace, animal fattening location, slaughterhouse, and cutting and boning room. Brazilian consumers were asked which of the two labels provided the necessary information to satisfy their requirements.

Finally, participants from both countries were asked if, in case of suspicion that the meat they purchased has a problem of food safety quality or other aspects related to health, what action would they take with respect to the use of that food.

### 2.4. Data Analysis

Statistical analyses were performed using IBM^®^ SPSS statistics version 22. Univariate and bivariate analyses were used to evaluate characteristics related to consumer behavior. Descriptive and chi-square analyses were used to evaluate characteristics related to consumer behavior: Knowledge, credibility, and importance of traceability and utility of information traceability on the beef label.

A maximum significance level of 5% or less was tested for acceptance or rejection of the null hypothesis. The conjoint analysis, using the dependency method, was used to determine the preference of different levels of Spanish beef label traceability information. As variables, the presence of mandatory traceability data, the presence of additional information and the presence of a QR code were analyzed, with two levels, omission/presence, in each variable.

## 3. Results and Discussion

### 3.1. Knowledge of the Traceability System for Beef

The use of traceability information at the time of purchase results depending on knowledge of the concept [35]. This investigation, in line with these findings, starts with an interest in understanding if Spanish and Brazilian consumers are familiar with traceability.

According to the results of this study, just over half of the Spanish consumer sample (52.8%) and 58.4% of Brazilian consumers say to be perfectly familiar with the concept of traceability. While about a third of consumers in both countries indicate they do not know traceability, the majority of respondents chose the correct definition of “monitoring of beef origin and products” representing 81.2% of Spanish consumers and 77.5% of Brazilians (Table 1).

Consumers with no traceability information end up not being interested in this subject [46]. In two studies evaluating knowledge of the concept of traceability, one in 2006 [47] and another ten years later, in 2016 [33], it was shown that considerable difficulty in hearing about traced meat was and continues to be associated with the lack of product availability and accessibility of product information.

It is worth noting that 11.4% of Brazilian consumers chose the traceability concept in this study: “Beef inspected by the health service” (Table 1). This choice is likely to refer to the quality inspection service for the production of food products of Brazilian animal origin (MAPA—Ministry of Agriculture, Livestock, and Supply) whose certificate seal is present in all packages of products of animal origin, with three levels of inspection: Federal Inspection Service (S.I.F), State Inspection Service (S.I.E) and Municipal Inspection Service (S.I.M) [48]. Our findings were confirmed by other studies carried out in Brazil that indicate that more consumers are aware of certificates of inspection for animal products than of traceability information or CoO [12,33].

For many consumers, the term that indicates information such as “monitoring” and “inspection” offers an additional impression of product protection, as is the case with traceability [33,46]. The results of this study suggest that consumers hesitated about the definition of traceability at first but once introduced to the definition, most will know how to identify it (Table 1).

### 3.2. Aspects that Make up the Current Traceability System for Beef

The ability to organize the chain efficiently is the basic requirement of the traceability system [47]. The introduction of traceability systems for the beef supply chain in some countries is based on mandatory legislation (such as Spain), while in others, regulations have been adopted on a voluntary basis, where market-dependent incentives for their implementation (such as Brazil) [33,47].

The first step towards the efficient use of traceability by consumers seems to be primarily related to informing consumers about the origin of the product, i.e., the various steps in which they show that the quality and safety of the product has been regulated [49].

Despite the different covering, one mandatory and the other voluntary, the regulations governing the traceability system in Spain and Brazil are the same. Consumers in both countries were therefore asked which aspects are incorporated into the existing traceability system. Table 2 shows a total of nine aspects, all of which are present in Spain and Brazil as part of the traceability system.

The findings indicate that more than half of the Spanish consumers surveyed identified only four aspects: The breed of the animal (61.5%), the date of birth (64.9%), the date of slaughter (71.3%), and the slaughterhouse (65.4%) (Table 2). From these results it can be concluded that the information that tends to be more common to Spanish consumers that is part of the traceability system is precisely the mandatory reference information found on the beef labels in this region, which are: Slaughter place and animal category of sales denomination provided for in Royal Decree 75/2009 [43].

A study has shown that specific aspects linked to the traceability system are less relevant for the general public/consumers and much more of interest to other market segments, such as producers, processors and the retail chain [47]. The stages of the process, which is quality related to safety which is a potential attribute of credibility to be valued at the time of purchase, are not evaluated out of their own, but what results from all of this [17,18].

In Brazil, more than half of consumers (45.5%) did not select just one attribute, “cutting room” (Table 2) The results found that the majority of Brazilian consumers are informed of the aspects that are part of the traceability system, according to researchers [50], because the information is known. This can be an indication that investments in the meat chain may bring benefits to retailers who need protection against the loss of consumer confidence.

### 3.3. Credibility and Importance of Traceability of Beef

The level of credibility and importance of beef traceability was evaluated in this study and the results (Table 3) showed that the credibility of traceability information is partial for 47.7% of Spanish consumers and 62.0% of Brazilians, whereas a greater proportion of Spanish consumers believe traceability information totally (28.0%) than Brazilians (14.6%). Spanish consumers also rely more than Brazilians on information such as: Origin and production of animals, marketing information, information on the slaughterhouse, and information on the animal itself.

Our study suggests that the fact that traceability information is provided more confidence by Spanish consumers than by Brazilians come from the fact that the traceability and labeling system in Spain is mandatory, and mainly because it is already consolidated, unlike in Brazil, where both traceability and labeling systems are voluntary, often only for marketing purposes, as some researchers suggest [37].

According to previous research [18,49], the credibility of traceability information is determined by its accessibility and the consumers’ own reasoning. In order to become an attribute of confidence for the user, traceability information needs to be driven by the advantages that this information brings [27].

As far as the importance of the traceability system is concerned, more Spanish consumers (59.6%) and to a minor extent, Brazilians (50.9%) agree that traceability is important to consumers, that is to say, to themselves. To a minor extent, about a third of Spanish consumers (33.7%) and Brazilians (31.1%) agree that traceability is important for companies (Table 3). According to some researchers [13,18], although consumers partially believe in traceability information, they still believe that traceability remains important for them, data that corroborate our study.

A study conducted with Brazilian consumers [27] confirms the importance of attributes such as traceability in the decision-making process for purchasing beef. Other research, also carried out in Brazil [33,51] found that the majority of beef consumers, more than 90% of them, agreed that meat with any kind of certification has greater benefits than meat without any certification, and consumers believe that certificate products are better, higher quality and more secure.

Knowing the perceptions and requirements of consumers about food traceability is often an obstacle, because credit attributes can be interpreted with different ways, such as marketing purposes for example. Misinterpretations can lead consumers not to know the real reason for traceability, which is to improve food security against hazards that may occur at different points in the food chain [37,52].

For this reason, industry and government must focus their efforts to ensure that messages are properly transmitted and understood by consumers from credible attributes, such as traceability information [17,37].

In general, measures to minimize consumer inaccurate vision are straightforward and efficient communication, that is to say, in a format that is easily accessible and without overloading consumers. This suggests more visible labeling with certification assurances during buying, giving customers the ability to more clearly evaluate meat security [53].

### 3.4. Usefulness of Traceability Information on Beef Labels

Food labels aim to enable consumers to make informed decisions about experience and credibility attributes such as product quality, technology for production, and processing [52,54]. Given that consumer perceptions of food safety risk are strongly linked to the credibility of product attribute information, food labeling regulation needs to ensure the integrity of that information [37].

The information on the meat label is considered to have higher or lower levels of importance at the time of purchase. Research performed with meat consumers indicate that readily interpretable information, such as meat type/cut and expiration data, has a higher rating of significance and use compared to credit information indications, such as origin [17,46,47,55].

Our study evaluated the utility for Spanish and Brazilian consumers of traceability and/or CoO information on beef labels. Since traceability information is obligatory on beef labels in Spain and on a voluntary basis in Brazil, concerns about the utility of this information have been handled in accordance with the reality of the consumers in question (Table 4 and Table 5).

Spanish consumers were initially asked if they had taken the traceability information on the packaging labels into account while purchasing beef (Table 4). Most respondents said yes (57.3%) compared to 42.7% who said that they did not. The results also showed that most Spanish consumers use beef traceability information to avoid purchasing products from unknown or suspicious countries (32.7%) or just want to know the origin of meat (22. 9%). Beef originating in the country itself (20.7%), their region (13.0%), or the European Union (EU) (10.6%) are preferred, respectively.

Consumers who do not take traceability into account at the time of purchase are mainly due to never having been concerned with such information (31.3%), or because they do not know what to do with this traceability information (21.3%), for knowing that it is tracked is sufficient (19.6%), for thinking that this information is not their responsibility (11.7%), for not having time to read (4.9%), or not seeing any use of the traceability information for beef (2.4%) (Table 4).

In the EU, due to concerns on BSE, mandatory traceability information on labels for beef was introduced in 1997 in order to be used by consumers to infer product quality [56]. It has been shown [40,49] that the mandatory CoO benefits the domestic market in general if the preference for national products over imported products is high enough. According to our results, this is what, is happening within the Spanish market.

Also, according to a research on red meat in Spain [57], the origin of the meat was the most important factor in determining the consumer’s purchase decision and one of the most important for German and Polish consumers in the decision to purchase of pork [26]. However, as other researchers have found out [46,58], the information indicating the product’s visual quality is often more important than the signs on the labels relating to traceability and origin.

Other studies on the importance attributable to CoO information on the label [59] show that consumers can be pessimistic about this information because they do not believe, do not see advantages in the information offered, and do not want to pay for an attribute that does not value sufficiently.

In this research we found that traceability information is useful (of usefulness) to consumers who do not feel responsible for the label’s traceability information, or do not know what to do about it (commitment and lack of knowledge); when purchasing beef. Other researchers [13] also indicate that consumers are not interested in the information on traceability itself, but in knowing that traceability has been established and that someone is inspecting the background of the meat. According to a study by Belgian consumers [47], about 70% of respondents prefer retailers or butchers to store all the necessary traceability information and to make it available at the consumer’s request.

In Brazil, labeling with CoO information on beef is voluntary, and because not all establishments supply beef with this information, Brazilian consumers were asked whether they had ever bought beef with CoO information on the label.

We found that 45.3% of consumers say they do not know whether they have bought beef with CoO or not. Another 32.1% said yes, having already bought beef with CoO against 22.6% that said no (Table 5). A 46.4% of the customers who said they bought beef with CoO had previous knowledge of traceability from other products, and 31.7% did so by observing the CoO information provided on the label for themselves, another 17.1% due to marketing at the establishment or other communication channels, and 4.8% due to interaction/conversation with other individuals having been informed about beef CoO (Table 5).

On the other hand, in the case of consumers who said they had never bought beef with CoO on the label, the large majority (84.6%) of consumers said they did not know where it was sold and/or believed it was not available at their place of purchase; 9.1% do not know where to sell, nor are they interested in purchasing in the same way and 6.3% say they do not buy because they do not see a difference between a product with or without traceability or certification of origin (Table 5).

In a study conducted in Brazil [12], it was found that most consumers would like to have access to information on meat traceability and would be willing to pay more for this product, supporting Brazil’s mandatory meat traceability. CoO beef products are gaining place on the shelves of large beef distribution chains, but the supply of these items is still limited, in most cases, to the packaging of vacuum-packed whole cuts [33].

In relation to the credibility and origin of beef, a vast literature on consumer preferences and on willingness to pay more for labeling initiatives is available [28,33,52,59]. The question is to what degree consumers actually value this information, considering the increasing use of CoO information on food labels [60,61].

The challenge, then, is how to communicate the use of food security technologies as consumers are willing to pay for these technologies [62]; and the best way has been to effectively label food [63].

The best way to provide this information on food labels is to confront costs vs. consumer attitudes/perceptions through the demand for origin information of the product, taking into account the costs of mandatory implementation of traceability or CoO, which are high [47,52]. Currently, the findings most commonly seen in research studies [61] are that consumer expectations for origin knowledge for meat products are clearly growing.

The usefulness of the traceability information for consumers depends on how far the traceability situation is in the country and the contribution of consumers’ knowledge on this matter. For example, it was found that consumer risk perceptions in the US and Japan [52] are significantly affected more by their level of confidence in credit attribute information such as organic, natural, traceable, and country of origin, than by credibility in visual attributes such as color and texture.

Studies conducted in countries that do not have a mandatory traceability system until then, as in China [64] and Poland [65], indicate that regular meat consumers would benefit from the introduction of this system because this information could serve as a cognitive shortcut in evaluating the quality of beef and mechanisms of food safety in the public health perspective.

These findings are relevant to industry and policy, as they indicate that more advanced animal information quality tips give a sense of control throughout the food chain and help consumers infer the credibility of products [18,38].

Consumers can look for and expect more information about the CoO of a product, but if consumers do not value it sufficiently, there is no reason to force the provision of this information [66]. This does not mean that they are irrelevant, although not of general relevance to consumers, but from an informative research perspective that should be used to target specific consumer segments rather than all consumers [38].

### 3.5. Preference for Traceability Information on the Label for Beef

In order to satisfy consumer needs, the traceability information must be in the format desired by the user and capable of understanding their needs [52]. Preferences for beef products may not be similar among consumers in different countries [67]. Therefore, the preferences for how traceability and/or CoO information is presented on the beef label by Spanish and Brazilian consumers were also evaluated in this study (Table 6, Table 7 and Table 8).

First, Spanish consumers were questioned about the need for changes in the current display of traceability information on beef labels (Table 6). The results show that the information provided on labels is sufficient for approximately 48% of Spanish consumers interviewed and do not see the need for adjustments. Another 36% of consumers agree that the beef label does not require codes or indications to prove that this product has been traced. About 13% of consumers said that the traceability information on beef labels would be better represented using a barcode/QR code.

Six labels with different levels of traceability information have been presented, going deeper into preferences in the way in which beef traceability information is presented on labels to Spanish consumers (see set of labels Appendix A).

Regarding the preference for labels which present “traceability” information, the preference is higher, followed by labels with only “basic” product information. For Spanish consumers, labels with additional information on ‘plus’ traceability are not necessary. The results (Table 7) show that information written in the label (75.4%) is more important than that transmitted by a QR code (24.6%), in other words, the omission of the QR code is preferred.

The results suggest that Spanish consumers already seem to be used to the amount and way in which the traceability information is passed to them through the labels, because a large proportion of consumers believe that there is no need to modify the label and that the current available information is satisfactory. It is noted that Spanish consumers are satisfied with how traceability information is made available, without needing to add codes/symbols or even extra information. Also, the lack of information on traceability is not well accepted (Table 7).

In a similar study [47] which confronted consumers with different levels of label traceability information, the most preferred label was the one with traceability information on it. Participants also largely rejected the meat label without concrete information on traceability. In general, the results are indicative that a direct reference to origin may be an effective option when it comes to informing consumers about the traceability of meat.

It can be assumed at the time of purchase that consumers prefer a simpler presentation of traceability system information, that is, easy to understand and that gives them direct access to the information they need or prefer [68].

Studies [49] have shown a limited consumer interest in providing traceability information by codes, such as Radio Frequency Identification (RFID) tags (which have now been replaced by QR code), and a greater preference for simple labels or information written on product labels. This information corroborates the findings in this study.

In Brazil, in order to determine the preference for the display of beef traceability information on the labels, two labels were filed: One of them was a reproduction similar to that of a beef label sold to Spanish consumers (same traceability information) and the other was with information such as that given by most products traced in Brazil (traceability number of animal) (see labels in Appendix B).

The findings (Table 8) show that traceability information on beef labels would be better represented for 56.7% of the consumers questioned if it were a combination of labels from both countries i.e., with information available at the time of purchase (Spanish label) and also with access at any time to the origin of the product by purposes of a QR code (Brazilian label). The second opinion with the highest number of responses was from consumers who prefer the Spanish label (31.6%) versus 10.4% who prefer the Brazilian label and 1.3% who do not prefer any of them, saying that the label does not require any traceability information.

In an experiment [61], in which the willingness of the consumer to pay for CoO information on meat and meat products was investigated, it was shown that 41, 7% of participants preferred labels with a text and a symbol together, which was moderately more than just indicating with a symbol (40.8 percent), and 17.5% preferred text only.

Preferences on how the label should be interpreted with traceability information are a somewhat contentious subject. As researchers [31,35] say, consumers need simple and concise information because of the limited time available to purchase, but at the same time they will be interested in obtaining more elaborate and accurate information, that could be, such as through the Internet for example.

Other studies [28] have described that the greater the quantity of label information, the higher the purchase of a product.

Researchers [34] clarify more specifically that the preferences for any label information depend on the type of product under consideration. For instance, when products are new and unfamiliar, as well as fresh products, more detailed information is required, while concise information would be appropriate for regular purchases or non-perishable products.

### 3.6. Suspected Food Security Questioned

Food safety differs from other attributes of quality because it is a hard aspect to observe. A product may appear to be of high quality, but it may not be safe because it may be contaminated and go unnoticed before the product is ingested and the effects are manifested [13].

For this reason, food producers and policy makers are of particular interest in the consumer response to food safety information, then we investigate the attitude of consumers when there is a suspicion that purchased beef has a safety risk, quality, and other aspects (Table 9).

The majority of surveyed Spanish consumers (72.5%) and 41.5% of Brazilians answered that they do not use the product and throw it away when faced with a potential food danger. More than half (53.2%) of Brazilians claim, that in addition to not using meat, they call the customer information phone number available on the label. A minority of 8.9% of Spaniards and 5.3% of Brazilians use meat because quality and safety are guaranteed due to the fact that it is monitored/traced (Table 9).

The results show that more Brazilian consumers report concerns to those responsible for the product than Spanish consumers (Table 9).

According to researchers, the most immediate reaction when there is suspicion regarding safety and quality is not to buy the product again. Of the product in hand, that is, already purchased, most consumers throw away or call an information telephone, such as government agencies and consumer organizations, as well as complain directly to the retailer, which is called “complaint behavior”. A minority uses the justification that, because it is traced, the safety and quality of the meat are guaranteed and can be eaten [34,35].

Traceability can have a tremendous value when talking about prevention in potentially hazardous products, such as meat, that lead to serious health consequences when contaminated [37].

Recalls are the main means by which consumers are warned that certain products are contaminated with harmful substances or microorganisms. Robust evidence has been found that a change in the way people interpret and react to beef recalls was induced by BSE cases in 2003 [35].

When consumers begin to respond to recalls, this means that consumers reject contaminated meat, there is an increase in purchases from companies that do not involved in the recall, which provides incentives for the beef industry to work with more control guarantees for reduced recalls and companies suffer less damage [35]. When everybody, and not just the industry, has access to information about the origin and quality of the product, the relationship between the parties (consumers and the supply chain) works best. This improves the brand name and increases its market credibility [37].

Currently, the attribute that people most associate with their own words to traceability is recall. However, consumers will only benefit from this information when it is reliable, otherwise consumers will likely lose confidence in the traceability system and the information it provides [34].

## 4. Conclusions

This cross-cultural research study aimed to compare Spanish and Brazilian consumers with the purpose to understand the familiarity with the bovine traceability system and the label traceability information as an indicator of product safety in countries with different beef consumption, production, and traceability system implementation.

Consumers do not have a clear understanding of the term “traceability systems” but may be able to indicate the utility of such a system. In the role of guiding and to be able for consumers understanding traceability, more effective public policy may help to explain and clarify the general benefits associated with the purchase of food with traceability certified.

It seems that Brazilians have more technical knowledge of the traceability system and its component aspects than Spanish consumers. It may be because of Brazilians’ familiarity with the production of animals, since the country is an important producer/consumer of beef. On the other hand, Spanish consumers are less acquainted with the system itself, but far more familiar with the knowledge that reaches them via the labeling.

It is also clear that since traceability and labeling are mandatory in Spain, consumers have more credibility and offer the traceability system more importance, this can be a factor that can be taken into account in the decision-making process of a company looking to implement traceability where traceability is not established.

Consumers are interested in making sure that the item they purchase is of known and trusted origin. Consumers who do not take traceability information into account at the time of purchase are not feel responsible for traceability because they lack knowledge and are not engaged in product information. The implementation of a mandatory traceability system would only be interesting if consumers value this information. Again, public incentives would be a way to increase consumer demand for security.

With regard to the presentation of label traceability information, Brazilian consumers prefer to use combined information in the form of text and symbols (QR code). Traceability information in text form is well accepted by Spanish consumers. There is no successful acceptance of information provided with a QR code or an excess of written information.

Brazilian consumers are more concerned with reporting a potential risk in food, this may be an indicator that Brazilians will be more involved with respect to “complaint behavior” with compulsory traceability and this would bring benefits to public health in the case of any food issue that might arise.

## Figures and Tables

**Table 1:** Knowledge and definition of the traceability concept for Spanish and Brazilians consumers.

Traceability Concept *	Country ^1^		*p*
	Spain	Brazil	
Knowledge of the concept			
Yes, perfectly	52.8	58.4	0.045
No, totally unaware	36.0	33.3	
Unclear	11.2	8.3	
**Definition of the concept**			
Monitoring of beef origin and products	81.2	77.5	≤0.001
Beef inspected by the health service	3.4	11.4	
Nutritional information by labels	2.3	1.3	
No chemical residues contaminants in beef	0.7	0.4	
Branded beef	0.5	0.8	
Don’t know	11.9	8.6	

^1^ Total *n* = 2132, Spain *n* = 436, Brazil *n* = 1696. * Only one answer per question. (%).

**Table 2:** Aspects assumed by Spanish and Brazilian consumers are part of the current traceability system for beef.

Aspects of Traceability System *	Country ^1^		*p*
	Spain	Brazil	
Animal feed	49.5	75.9	≤0.001
Animal breed	61.5	76.2	≤0.001
Date of birth	64.9	78.9	≤0.001
Animal birthplace	48.4	72.4	≤0.001
Animal fattening location	49.3	75.5	≤0.001
Transport	44.7	73.1	≤0.001
Slaughter date	71.3	86.9	≤0.001
Slaughterhouse	65.4	87.7	≤0.001
Cutting and boning room	37.6	45.5	0.003
Don’t know	17.2	4.6	≤0.001

^1^ Total *n* = 2132, Spain, *n* = 436, Brazil, *n* = 1696. * Multiple answers possible. (%).

**Table 3:** Credibility and importance in the traceability information that Spanish and Brazilian consumers believe is appropriate to their way of thinking.

Credibility and Importance of Traceability *	Country ^1^		*p*
	Spain	Brazil	
Total credibility	28.0	14.6	≤0.001
Partial credibility	47.7	62.0	≤0.001
No credibility	7.3	2.9	≤0.001
Credibility in the origin and production of animal	33.7	16.6	≤0.001
Credibility in marketing information	24.8	7.3	≤0.001
Credibility in slaughterhouse information	19.0	8.8	≤0.001
Credibility in the information of the animal itself	13.5	6.3	≤0.001
Traceability is important for consumers	59.6	50.9	0.001
Traceability is important for companies	33.7	31.1	0.301

^1^ Total *n* = 2132, Spain *n* = 436, Brazil *n* = 1696. * Multiple answers possible. (%).

**Table 4:** Opinion of Spanish consumers on information related to the traceability in beef labels.

Traceability of the Beef Label	Spain ^1^
Do you take into account the data related to traceability on beef labels?	
Yes	57.3
No	42.7
In case of AFFIRMATIVE answer ^2,^*	
I don’t buy beef from unknown or suspicious countries	32.7
I want to know the origin of the product	22.9
I only buy beef originating from my country	20.7
I only buy beef originating from my region	13.0
I only buy beef from the EU	10.6
In case of NEGATIVE answer ^3,^*	
It has never worried me	31.3
I don’t know what to do with that information	21.3
Knowing there is traceability is enough	19.6
Traceability code must be on the products, but it is not my responsibility	11.7
This information is difficult to interpret	8.7
I don’t have time to read it	4.9
I don’t see the use of it	2.4

^1^ Total *n* = 436, ^2^ only for people who said “yes” *n* = 250, ^3^ only for people who said “no” *n* = 186. * It is possible to mark several points. (%).

**Table 5:** Opinion of Brazilian consumers on information related to the traceability in beef labels.

Traceability of the Beef Label	Brazil
Do you buy or have you already bought beef with traceability information on the label?^1*^	
Yes	32.1
No	22.6
I don’t know if I bought/didn’t realize it at the time of purchase	45.3
If YES, how did you hear about this product?^2*^	
Knowledge of the traceability of agricultural products	46.4
Observing the identification of traceability on the label	31.7
Marketing in the establishment where I buy or in other channels of communication	17.1
In conversations with others	4.8
If NO, why never bought it? ^3*^	
I don’t know where it sells/I think it’s not available in my city	84.6
I don’t know where it sells and I’m not interested in buying it	9.1
I don’t buy it because I don’t see a difference between meat with traceability or without it	6.3

^1^ Total *n* = 1696; Minas Gerais, *n* = 424; Sao Paulo; *n* = 456, Parana, *n* = 406; Santa Catarina, *n* = 410. ^2^ Total *n* = 545 (only for people who said “yes”): Minas Gerais, *n* = 125; Sao Paulo, *n* = 161; Parana, *n* = 141; Santa Catarina, *n* = 118. ^3^ Total *n* = 383 (only for people who said “no”): Minas Gerais, *n* = 127; Sao Paulo, *n* = 70; Parana, *n* = 90; Santa Catarina, *n* = 96. * Mark only one answer. (%).

**Table 6:** Opinion of Spanish consumers according to changes in the traceability information on beef label.

Changes in Traceability Information on Beef Labels *	Spain ^1^
No need changes	47.9
No need for codes or symbols	36.0
Better represented by barcode/QR code	13.1
Don’t know	3.0

^1^*n* = 436. * Mark only one answer. (%).

**Table 7:** Spanish consumers’ preference according to the amount of information on the beef label.

Attributes	Importance Values ^1^	Levels	Estimated Preference	ERROR	*p*
QR Code ^2^	24.6	Yes	−0.317	0.142	≤0.001
		No	0.317		
Information ^3^	75.4	Basic	0.226	0.201	
		Traceability	1.198		
		Plus	−1.424		

^1^*n* = 436. ^2^ “QR code”: Module to store information in a dot matrix or in a two-dimensional barcode. ^3^ Information – type of information on the label: “Basic”: Name of the product, the category, the recommendations for cooking, the expiration date, the price, etc. “Traceability”: Traceability number of the animal, animal birthplace, animal fattening location, slaughterhouse and cutting and boning room. “Plus”: The “Traceability” information mentioned above, with the addition of information on: Breed, sex and date of birth of the animal.

**Table 8:** Brazilian consumers’ preference according to the amount of information on the beef label.

Display of Traceability Information on Beef Labels *	Brazil ^1^
I prefer it to be a combination of the two labels, with information I can see at the time of purchase (Spanish label) or that I can access it over the internet if I’m interested in product origin (Brazilian Label)	56.7
I prefer the Spanish label, because it brings traceability information in the product label at the time of purchase	31.6
I prefer the Brazilian labels, if I’m interested in knowing the information of traceability I can access it over the internet through my cell phone or computer at moment or after purchase	10.4
None of the labels, I don’t see the need for traceability information for beef	1.3

^1^ Total *n* = 1696; Minas Gerais, *n* = 424; Sao Paulo, *n* = 456; Parana, *n* = 406; Santa Catarina, *n* = 410. * Mark only one answer. (%).

**Table 9:** Consumer behavior when beef is suspected of presenting a problem in terms of safety, quality and other aspects.

Attitude of the Consumer Towards Suspect Beef Quality *	Country ^1^		*p*
	Spain	Brazil	
I don’t use it and throw it in the trash	72.5	41.5	≤0.001
I don’t use it, but I keep it and call customer service phone number	18.6	53.2	
I use it because safety and quality are guaranteed by the producer	8.9	5.3	

^1^ Total *n* = 2132, Spain *n* = 436, Brazil *n* = 1696. * Mark only one answer. (%).

**Figure A1:**
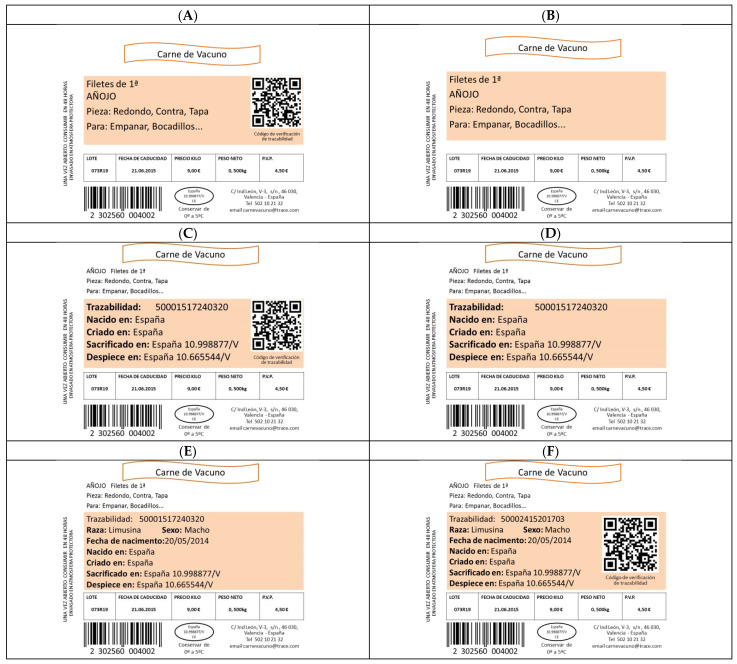
(**A**)—Traceability information available on the label: QR Code. (**B**)—Label without traceability information available. (**C)**—Traceability information available on the label: Written information (provided for by Royal Decree 75/2009) and QR Code. (**D**)—Traceability information available on the label: Written information (mandatory provided for by Royal Decree 75/2009). (**E**)—Traceability information available on the label: Written information (provided for by Royal Decree 75/2009) with additional information added by the authors. (**F**)—Traceability information available on the label: Written information (provided for by Royal Decree 75/2009) with additional information added by the authors and QR Code. Information contained within the colored card: Trazabilidad: (traceability number); Raza: (Animal breed); Sexo: (Gender); Fecha de nacimiento: (Date of animal birth); Nacido en: (Animal birthplace); Criado en: (Animal fattening location); Sacrificado en: (Slaughterhouse); Despiece en: (Cutting and boning room); Código de información de trazabilidad: (QR code).

**Figure A2:**
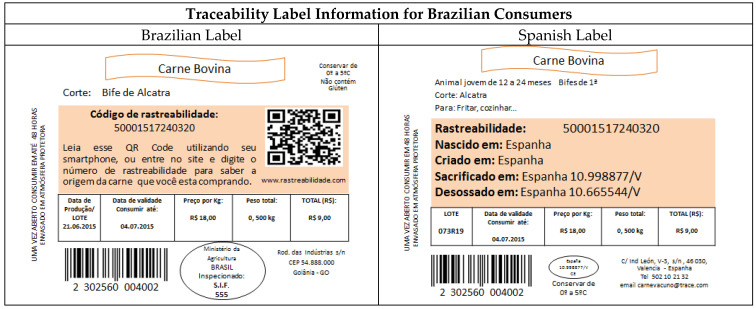
Information contained within the colored card: Código de rastreabilidade: (Traceability number); QR code; Rastreabilidade: (traceability number); Nascido em: (Animal birthplace); Criado em: (Animal fattening location); Sacrificado em: (Slaughterhouse); Desossado em: (Cutting and boning room).

## Supplementary Materials

The following are available online at https://www.mdpi.com/2304-8158/10/2/232/s1, Annex 1: Self-administered questionnaire.

## Appendix A

**Table A1:** Distribution of traceability information on each of the labels presented to Spanish consumers.

Label	Traceability Label Information for Spanish Consumers		
	Traceability	Extras (Plus)	QR Code
A			x
B			
C	x		x
D	x		
E	x	x	
F	x	x	x

x – presence of information
