# Supplementary material for: Knowledge, Utility, and Preferences for Beef Label Traceability Information: A Cross-Cultural Market Analysis Comparing Spain and Brazil
Source: Foods. 2021 Jan 23;10(2):232. doi: 10.3390/foods10020232 (PMC7911574; doi:10.3390/foods10020232)
Supplement: Supplementary file 1 [file foods-10-00232-s001.pdf]

Annex 1 - Self-administered questionnaire

Common questions

1-You know the concept of traceability, especially with respect to beef?

- Yes, perfectly
- No, totally unaware
- Unclear

2- In your opinion, the concept of meat traceability can be described as:

- Meat with monitoring of the origin of production and intermediate processes until the final supplier
- Meat inspected by the health service
- The meat that has nutritional information on the label
- Meat that has no contaminants or chemical residues
- Branded meat
- Do not know

3- What aspects do you believe are part of the traceability system?

- Animal feed
- Animal breed
- Date of birth
- Animal birthplace
- Animal fattening location
- Transport
- Slaughter date
- Slaughterhouse
- Cutting and boning room
- I don't know any aspect

4- What is your level of confidence concerning beef traceability?

- Total, is fully controlled by health authorities
- Partial, this is a very complicated process that can suffer errors
- No credibility
- It only inspires confidence regarding the origin and production of animals
- It only inspires confidence in the information about the animal itself
- It only inspires confidence in the slaughter of animals
- It only inspires confidence regarding the marketing information
- Traceability is important for the company
- Traceability is important for the consumer

5- In the suspicion that the purchased beef has some problem of safety, quality or other aspects, what is your attitude?

- I don't use it and throw it in the trash
- I don't use it, but I keep it and call the phone available on the customer service label
- I use it because safety and quality are guaranteed by the producer

Question only for Spanish consumers

6- Do you take into account the data related to traceability on beef labels?

- Yes
- No

7- In case of AFFIRMATIVE answer: - only for people who said yes

- I just want to know the origin of the product, but I don't care where comes.
- I only buy meat originating from my region
- I only buy meat originating from my country

- I only buy meat from the EU
- I don't buy meat from unknown or suspicious countries

8- In case of NEGATIVE answer: - only for people who said no

- It has never worried me
- Knowing that there is traceability is enough
- I don't know what to do with that information
- This information is difficult to interpret
- I don't see the use of it
- I don't have time to read it
- Traceability code must be on the products, but it is not my responsibility

9- Regarding the current information on traceability codes on beef labels:

- I don't see any need for change
- It would be better represented by a symbol e.g.: barcode/QR code, through which any other information could be accessed
- I don’t see the need for codes or symbols, the traceability information does not are important at the time of purchase
- Do not know

10- Six labels with different combinations of traceability information were displayed: from the most preferred to the least preferred labels on request. (Appendix 1)

Order the labels from most to least preferred:

- Most preferred
- 2nd most preferred
- 3rd most preferred
- 4th most preferred
- 5th most preferred
- Least preferred

Questions only for Brazilians Consumers

11- Do you buy or have you already bought beef with traceability information on the label? (Mark only one answer)

- Yes
- No
- I don't know if I bought/didn't realize it at the time of purchase

12- If YES, how did you hear about this product? Through... - only for people who said yes

- Marketing in the establishment where I buy or in other channels of communication
- Conversations with other people
- Observing the identification of traceability on the label
- Knowledge of the traceability of agricultural products

13- If NO, why never bought it? (Mark only one answer) - only for people who said no

- I don't know where it sells/I think it's not available in my city
- I don't know where it sells and I'm not interested in buying it
- I don't buy it because I don't see a difference between meat with traceability or without it

14- Regarding the current traceability information on beef labels: (Appendix 2)

- I prefer the Brazilian labels, if I’m interested in knowing the information of traceability I can access it over the internet through my cell phone or computer at moment or after purchase
- I prefer the Spanish label, because it brings traceability information in the product label at the time of purchase
- I prefer it to be a combination of the two labels, with information I can see at the time of purchase (Spanish label) or that I can access it over the internet if I'm interested in product origin (Brazilian Label)
- None of the labels, I don't see the need for traceability information for beef
